# CMCompare webserver: comparing RNA families via covariance models

**DOI:** 10.1093/nar/gkt329

**Published:** 2013-05-02

**Authors:** Florian Eggenhofer, Ivo L. Hofacker, Christian Höner zu Siederdissen

**Affiliations:** ^1^Institute for Theoretical Chemistry, University of Vienna, Währingerstrasse 17, A-1090 Vienna, Austria and ^2^Bioinformatics and Computational Biology Research Group, University of Vienna, Währingerstrasse 17, A-1090 Vienna, Austria

## Abstract

A standard method for the identification of novel non-coding RNAs is homology search by covariance models. Covariance models are constructed for specific RNA families with common sequence and structure (e.g. transfer RNAs). Currently, there are models for 2208 families available from Rfam. Before being included into a database, a proposed family should be tested for specificity (finding only true homolog sequences), sensitivity (finding remote homologs) and uniqueness. The CMCompare webserver (CMCws) compares Infernal RNA family models to (i) identify models with poor specificity and (ii) explore the relationship between models. The CMCws provides options to compare new models against all existing models in the current Rfam database to avoid the construction of duplicate models for the same non-coding RNA family. In addition, the user can explore the relationship between two or more models, including whole sets of user-created family models. Visualization of family relationships provides help in evaluating candidates for clusters of biologically related families, called clans. The CMCws is freely available, without any login requirements, at http://rna.tbi.univie.ac.at/cmcws, and the underlying software is available under the GPL-3 license.

## INTRODUCTION

In the past years, and especially with the development of high-throughput methods like RNA sequencing, the scientific community became more and more aware of the importance of non-coding RNAs. These transcripts are found in all domains of life and regulate essential pathways and cellular processes.

Homologs of known RNA sequences can be detected in genomes using a number of methods. For close homologs, sequence-based methods like Blast (1) provide an extremely efficient search method. More remote homologs accumulate mutations on the sequence level, whereas the structure tends to be conserved. In structural non-coding RNAs, most of the statistical information appears to be available with the sequence and secondary structure. Methods like Infernal (2,3) can be used to transform the structural alignment of an RNA family of related sequences into a stochastic model called a covariance model.

RNA family models allow one to find new homolog family members by considering the structure and sequence features of this family. The number of covariance models, which is available from databases like Rfam (4,5), is constantly increasing.

Putative homologs discovered in a genome should, in principle, show strong affinity to only a single RNA family or, by extension, covariance model. In practice, some RNA families [e.g. RNaseP, rRNA (SSU)] have been intentionally split along kingdoms to preserve statistical signals owing to diverse sequence mutations and structural changes.

The CMCompare webserver (CMCws) provides an easy-to-use interface to check the discriminatory power of newly proposed RNA family models. This makes it possible to check that a similar model does not already exist in the database or that a set of existing or newly proposed models is not too closely related to each other in terms of the sequences they accept as putative homologs.

## DESCRIPTION OF THE WEBSERVER

### Functionality of CMCws

For newly constructed covariance models, it is useful to check what other models are already available in Rfam and compare them with each other. The CMCws is based on ‘CMCompare’ (6), which returns a Link score for every pair of models checked. Link sequences and their associated Link scores are sequences giving high scores in both models simultaneously. A sequence with a Link score of, say 20 bits, scores at least 20 bits in each of the models. The Link sequence is the sequence with highest overall Link score (6). A high Link score can be an indicator for the following:

1. A model for the same RNA family is already present in the database. Using a curated model from Rfam avoids repetitive model construction and fine tuning. Also, improvements and extensions can be easier shared by finding and using a common set of models. Detection of a similar model by CMCws allows one to use this model instead.
2. At least one of the models lacks specificity, meaning that both score high for the same sequence. A model should detect only homologs belonging to the RNA family it represents, but not of member sequences of other families. During model construction, more members belonging to the RNA family are added to ensure detection over bigger phylogenetic distance, which can expand the space of detected sequences and associated structures to overlap with other families. By highlighting these overlaps, CMCws makes it possible to address this lack of specificity.
3. A biological relationship exists between the models that explain the overlap. Families derived from a common ancestor can share sequence and structure features. Rfam groups families related in this way as clans (7), which has been done up to now in a manual process. CMCws would allow Rfam to find possible candidates for clan members.

### Input

After choosing the mode of comparison, the web server accepts a file upload containing one or more Infernal covariance models (Infernal 1.0 or later, Rfam 9 or later) or structural alignments using the Stockholm format as input. Stockholm alignments are internally converted to covariance models for further processing.

### Processing

The web server relies on CMCompare (6), which is the first published tool for comparison of covariance models and has already been used in other projects (8,9). CMCompare has been expanded to also compare models created with Infernal 1.1 since publication.

Two modes of processing are available. The first mode allows one to compare the input models against all available models in Rfam or all models of specified subtype (micro RNAs, tRNAs) thereof, which reduces computation time. Alternatively, the set of uploaded models can be compared against each other.

### Output

**Figure 1.**
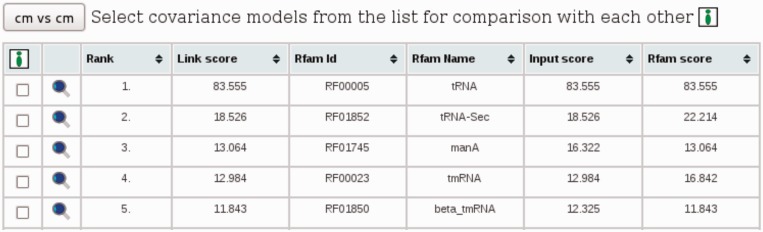
List of results: contains comparison results corresponding to the current filtering options. The list is sortable by all column names. The magnifying glass links to a detailed view of each comparison. The checkboxes on the right allow to select the models for a comparison with each other. CMCompare computes a score for the Input model (Input score) and for the Rfam model (Rfam score). The lower one is the Link score.

The first mode provides the user with a table of pairwise comparisons against Rfam models, as shown in Figure 1.

**Figure 2.**
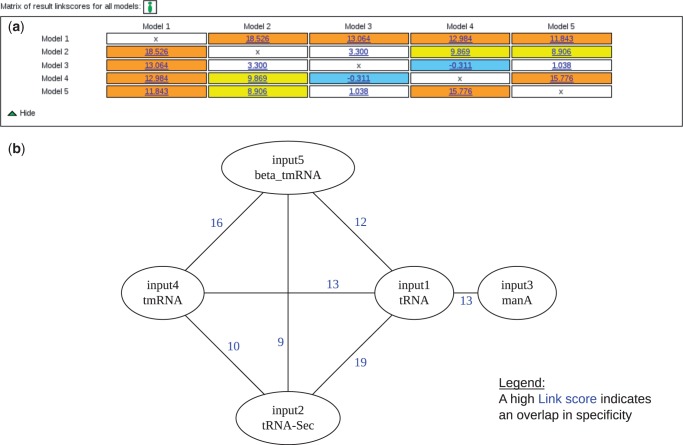
Visualizations: simplifying identification of relevant similarities between models by giving different representations of the pairwise result Link scores. (**a**) Link score matrix containing the similarity between all provided models and highlighting them by color. Clicking the Link score shows a detailed view of the comparison. (**b**) Weighted graph representation of linked models. The nodes indicate the models and contain their identifier. In contrast to the matrix representation, the shown edges correspond with the applied filtering options and redirect to a detailed view of the comparison on clicking. The comparisons against Rfam only show edges between the input and Rfam models. The shown input models 1, 2 and 4, 5 are members of the tRNA-clan, whereas ManA is presumably a false link.

The result list, computed by CMCompare, can be filtered by model name, Link score and number of models. Each of the columns can be sorted. These filtering options allow one to easily extract similar models. A weighted graph representation visualizes selected models as nodes, and their Link scores as edges to simplify evaluation, see Figure 2b. By clicking on the edges or the magnifying glass icon, each pairwise comparison can also be viewed in detail, providing the common highest scoring sequence (Link sequence), corresponding structure and further information.

Models of interest from the result list, or a set of models that have been uploaded by the user, can be analyzed with the second mode. This mode returns all pairwise comparisons, which can also be sorted and filtered by the name of a second model. Exploring this list is especially useful to identify groups of models that are closely related and pose potential candidates for clans.

The output is visualized as a graph, as well as a matrix, which gives all pairwise Link scores, simplifying the identification of relevant links, see Figure 2. Comparing models cannot always capture the biological relationship between models, e.g. in the RNase P clan. Although the two different models for bacterial RNaseP are linked with each other, one of them is strongly linked with the corresponding RNaseP model for archae, and the other one is not. By using a graph representation, we are still indirectly able to identify potential clan members.

As noted before, Rfam clans are constructed entirely manually. We believe that CMCws can significantly facilitate this process.

### Usage example

Assume we are interested in RNA families related with tRNAs. For this usage example, which follows Figures 1 and 2, we use as input the tRNA model (RF00005) from Rfam.

The first step is to select the comparison versus Rfam mode and upload the model to check for similar models already available from the database.

The top five resulting hits are shown in Figure 1, starting with tRNA having the maximal score possible with this model, compared with itself. The next models have Link scores between 10 and 20, indicating a moderate overlap between them.

For each of these models, one should investigate the reason for the high Link score, with potential reasons given previously as points 1–3. In decreasing order of Link score, we first consider the tRNA-Sec RNA family. Careful comparison of both secondary structures yields notable differences, including an additional stem in tRNA-Sec, but also some commonality. Based on commonalities and differences in biological action in the cell, as well as the differences in the structural alignment, one will probably not want to join both tRNA and tRNA-Sec, as a single family, but the commonalities are large enough to suggest a common Rfam clan, which for Rfam is true. Incidentally, the CMCompare algorithm proposes a consensus secondary structure of both RNA families for the link sequence, which contains a total of three stems, with one tRNA and two tRNA-Sec stems deleted in the consensus.

The next two models in the list tmRNA and beta_tmRNA have a significantly lower Link score than the tRNA compared with itself but capture the similarity between the models. As an aside, both tmRNAs have a higher Link score between each other than to the tRNA model.

The final model flagged by the CMCws is the manA RNA motif family. The Link score is low (13 bits) so that no immediate action is warranted.

However, the nature of the manA and its secondary structure (the CMCompare algorithm proposes a low-scored cloverleaf consensus structure between the tRNA and manA families) makes it a candidate for further investigation. According to Rfam, this is a computationally identified RNA family that occurs often adjacent to tRNAs (10).

Among the first five hits of the list, we can find three of the five other members of the tRNA clan. To get a better idea about their relationship with each other, we can select and resubmit them to a cm versus cm comparison. Figure 2b shows the result for the submission of the top five models. The matrix representation gives an overview over all comparisons between the submitted models, whereas the weighted graph only shows RNA family models as nodes and linkscores as edges. As expected, we can see that there is a strong connection between the members of this clan and especially between the tmRNA models. The manA is only linked with the tRNA model, but not with the other clan members. The combination of these two comparison modes simplifies finding candidates for clan construction.

Following these conclusions, the tRNA family would be submitted for inclusion in the Rfam database, pointing out it is possible biological relationship with the tRNA-Sec family.

### Implementation details

CMCws was implemented in Perl 5 using CGI.pm and the template toolkit. It relies on the jQuery library to allow sortable result tables. The underlying CMCompare algorithm (6) is implemented in Haskell (11). The conversion of input Stockholm-format alignments is done with cmbuild from the Infernal package (3).

The weighted graph representations of the output are created with dot from the graphviz (12) toolset.

The current version of the CMCompare algorithm has a quadratic runtime. With *n* and *m* the number of states (roughly the number of columns) in each covariance model, and *c* a fairly large constant, the runtime is 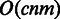. Wall-clock runtimes are from <1 s for small models to 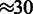 s for comparisons between members of the RNaseP clan. We plan to improve on these runtimes in the near future to facilitate large-scale comparisons.

### Other tools

To our knowledge, there are no algorithms available other than CMCompare that compare RNA family models with each other. Other classes of biopolymers like DNA or Proteins families can be modeled by profile hidden Markov models (HMMs) (13). General work has been done on comparing HMMs (14) with other HMMs. Also comparisons of HMMs with stochastic context free grammars (15), which provide the underlying principles of covariance models, have been investigated, but in both cases, no available tools originated from this work.

## DISCUSSION

CMCws simplifies dealing with an increasing number of RNA family models. Covariance models designed for essentially the same structural RNA family can be detected, as can those that capture a sub- or super-set of the structural features. Covariance models with inferior discriminatory power are easily detected by a large number of high Link scores to other RNA family models. Potential clans can be discovered by looking for a small set of CMs with higher Link scores to each other but low Link scores to all other families.

Challenges remain in identifying the cause of non-specificity among covariance models and how to defuse it. Suggestions how to split RNA families into more specific subfamilies and use of meta-families to pool them again could be a first step into this direction. Also, the construction of clans in an entirely unsupervised manner is a goal for the future.

Promising avenues for expanding functionality of CMCompare are other stochastic grammars such as HMMs used in Pfam (16).

This would allow expanding CMCws in the future to provide a comprehensive web server for comparing and analyzing different kinds of databases of stochastic sequence families.

## FUNDING

Austrian FWF, project ‘Doktoratskolleg RNA Biology W1207-B09’ (to F.E.) and project ‘SFB F43 RNA regulation of the transcriptome’ (CHzS). Funding for open access charge: Austrian FWF—SFB F43 RNA regulation of the transcriptome.

*Conflict of interest statement*: None declared.
